# Systemic Inflammation Disrupts Circadian Rhythms and Diurnal Neuroimmune Dynamics

**DOI:** 10.3390/ijms25137458

**Published:** 2024-07-07

**Authors:** Wai-Yin Cheng, Po-Lam Chan, Hang-Yin Ong, Ka-Hing Wong, Raymond Chuen-Chung Chang

**Affiliations:** 1 Department of Food Science and Nutrition, Faculty of Science, The Hong Kong Polytechnic University, Hong Kong SAR, China; hang-yin.ong@connect.polyu.hk (H.-Y.O.); kahing.wong@polyu.edu.hk (K.-H.W.); 2 Research Institute for Future Food, The Hong Kong Polytechnic University, Hong Kong SAR, China; po-lam-kathy.chan@polyu.edu.hk; 3 Laboratory of Neurodegenerative Diseases, School of Biomedical Sciences, LKS Faculty of Medicine, The University of Hong Kong, Hong Kong SAR, China; 4 State Key Laboratory of Brain and Cognitive Sciences, The University of Hong Kong, Hong Kong SAR, China

**Keywords:** circadian disruption, clock gene expression, microglia, peripheral circadian clocks

## Abstract

Circadian rhythms regulate physiological processes in approximately 24 h cycles, and their disruption is associated with various diseases. Inflammation may perturb circadian rhythms, though these interactions remain unclear. This study examined whether systemic inflammation induced by an intraperitoneal injection of lipopolysaccharide (LPS) could alter central and peripheral circadian rhythms and diurnal neuroimmune dynamics. Mice were randomly assigned to two groups: the saline control group and the LPS group. The diurnal expression of circadian clock genes and inflammatory cytokines were measured in the hypothalamus, hippocampus, and liver. Diurnal dynamic behaviors of microglia were also assessed. Our results revealed that the LPS perturbed circadian gene oscillations in the hypothalamus, hippocampus, and liver. Furthermore, systemic inflammation induced by the LPS could trigger neuroinflammation and perturb the diurnal dynamic behavior of microglia in the hippocampus. These findings shed light on the intricate link between inflammation and circadian disruption, underscoring their significance in relation to neurodegenerative diseases.

## 1. Introduction

Circadian rhythms regulate various physiological processes over approximately 24 h cycles. These rhythms are generated by a molecular clock mechanism involving interconnected transcriptional-translational feedback loops. The suprachiasmatic nucleus (SCN), located in the hypothalamus, acts as the master circadian pacemaker orchestrating oscillators within the central and peripheral tissues [1,2]. Both central and peripheral circadian oscillators are coordinated to modulate physiological and behavioral rhythms. At a molecular level, core clock genes include *Circadian locomotor output cycles kaput* (*Clock*), *Brain and muscle-Arnt-like 1* (*Bmal1*), *Period* (*Per*), and *Cryptochrome* (*Cry*) [3]. The transcription factors CLOCK and BMAL1 drive the rhythmic transcription of the *Per* and *Cry* genes. PER and CRY proteins then accumulate and form complexes that feedback to inhibit CLOCK-BMAL1 activity, repressing their own transcription. This intricate mechanism generates and regulates 24 h rhythms. Through the rhythmic control of downstream pathways, this molecular clockwork synchronizes internal physiological processes with external environmental cues. Disruptions of circadian rhythms are associated with various pathological conditions, including immune dysfunction [4,5,6,7], metabolic disorders [8,9,10,11], and neurodegeneration [12,13,14,15,16,17]. Circadian disruption can be induced by jet lag, shift work [18], misaligned light exposure [19], and other factors such as mistimed food intake [20] and medications [21].

Circadian clocks and the immune system are intricately interconnected, interacting bidirectionally at multiple levels. Circadian clocks are involved in modulating immune functions. Immune functions demonstrate diurnal oscillations, which is vital for regulating inflammatory responses and immunity maintenance [22,23,24]. The disruption of circadian rhythms can dysregulate immune responses [5,25]. On the other hand, the activation of the immune system may perturb circadian rhythms, disrupting physiological balance and manifesting in diseases [26]. While prior studies show circadian machinery modulates inflammation [22,23,24], whether inflammation disrupts circadian rhythms, particularly in the brain, remains unclear. Furthermore, our previous studies showed that systemic inflammation can provoke neuroinflammatory responses [27,28,29], activating microglia in the brain. This may interact with circadian regulation, as microglia exhibit diurnal rhythms in morphology under a normal physiological state [30]. The disruption of normal diurnal dynamics of microglia may affect neuroimmune responses. Studying diurnal microglia behavior and their morphology provides valuable insights into their functional state, which plays a crucial role in maintaining homeostasis of the central nervous system.

Here, we examined the effects of systemic inflammation induced by an intraperitoneal lipopolysaccharide (LPS) on the central and peripheral circadian rhythms, as well as neuroimmune dynamics. We hypothesize that systemic inflammation alters diurnal oscillatory patterns of core clock genes in central and/or peripheral circadian tissues and perturbs diurnal rhythms of microglial behaviors and expression of proinflammatory cytokine in the brain. This study aims to investigate the effects of systemic inflammation on circadian rhythms and diurnal neuroimmune activities.

## 2. Results

### 2.1. Intraperitoneal Injection of LPS Induced Systemic Inflammation and Alterations of Diurnal Expression of Proinflammatory Cytokines in Peripheral Tissue in Liver

**Figure 1:**
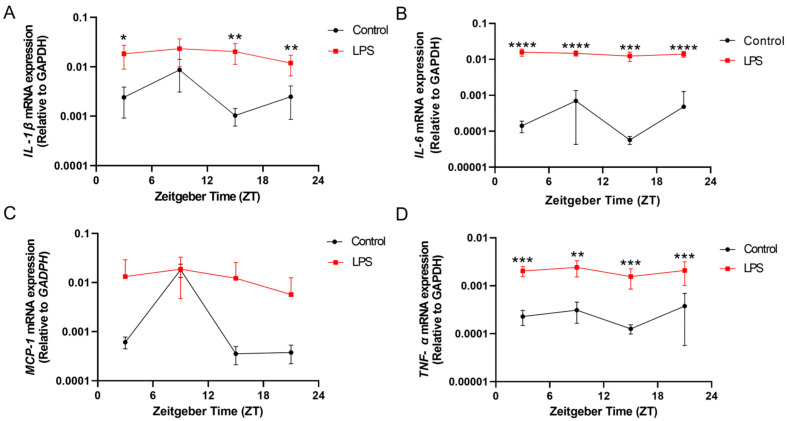
The intraperitoneal injection of an LPS induced systemic inflammation and perturbed the diurnal expression of proinflammatory cytokines in the liver. The mRNA expressions of (**A**) *IL-1β*, (**B**) *IL-6*, (**C**) *MCP-1*, and (**D**) *TNF-α* in the liver at 4 different time points (ZT 3, ZT 9, ZT 15, and ZT 21) were measured by RT-qPCR and relative to the endogenous gene *GAPDH*. Data are expressed as mean ± SEM. *n* = 3–5 mice per group for each time point. * *p* < 0.05; ** *p* < 0.01; *** *p* < 0.001; and **** *p* < 0.0001 by unpaired two-tailed Student’s *t*-test.

The intraperitoneal injection of the LPS induced systemic inflammation, as evidenced by the increase in proinflammatory cytokine expressions at different zeitgeber times (ZTs) on the second day post-LPS administration. At ZT 3, ZT 15, and ZT 21, a significant increase in *interleukin (IL)-1β* mRNA expression was observed in the liver of the LPS group when compared with the control (ZT 3: *p* < 0.05; ZT 15: *p* < 0.01; ZT 21: *p* < 0.01; Figure 1A). Both *IL-6* and *tumour necrosis factor-alpha* (*TNF-α*) mRNA expressions were also markedly elevated in the liver of the LPS group throughout the day, across multiple time points, ZT 3, ZT 9, ZT 15, and ZT 21, when compared with the control counterpart (*IL-6*: ZT 3: *p* < 0.0001; ZT 9: *p* < 0.0001; ZT 15: *p* < 0.001; ZT 21: *p* < 0.0001; *TNF-α*: ZT 3: *p* < 0.001; ZT 9: *p* < 0.01; ZT 15: *p* < 0.001; ZT 21: *p* < 0.001; Figure 1B,D).

Moreover, *monocyte chemoattractant protein-1* (*MCP-1*) mRNA expression showed significant diurnal rhythmicity in the liver of the control group (*p* < 0.05, Table S1 and Figure 1C). However, this rhythmic expression pattern was disrupted in the LPS group. These findings demonstrate that LPS administration abolished the circadian oscillation of the hepatic *MCP-1* mRNA level.

### 2.2. Systemic Inflammation Induced by LPS Resulted in Neuroinflammation and Alterations of Diurnal Expression of Proinflammatory Cytokines in Both Central Circadian Tissue in Hypothalamus and Peripheral Tissue in Hippocampus

**Figure 2:**
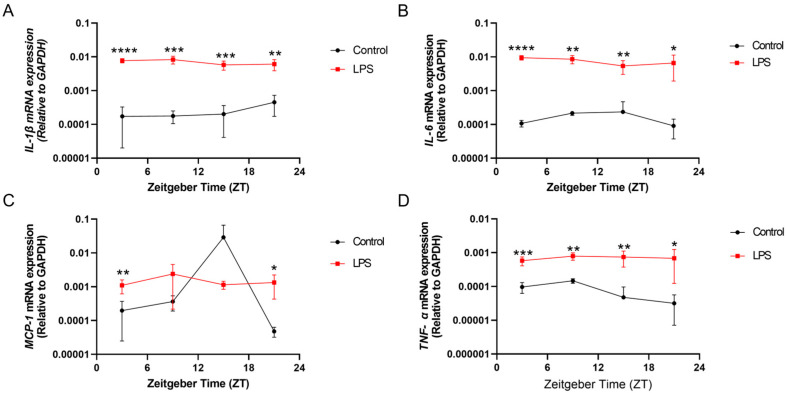
The intraperitoneal injection of an LPS induced systemic inflammation and perturbed the diurnal expression of proinflammatory cytokines in the hypothalamus. The mRNA expressions of (**A**) *IL-1β*, (**B**) *IL-6*, (**C**) *MCP-1*, and (**D**) *TNF-α* in the hypothalamus at 4 different time points (ZT 3, ZT 9, ZT 15, and ZT 21) were measured by RT-qPCR and relative to the endogenous gene *GAPDH*. Data are expressed as mean ± SEM. *n* = 3–5 mice per group for each time point. * *p* < 0.05; ** *p* < 0.01; *** *p* < 0.001; and **** *p* < 0.0001 by unpaired two-tailed Student’s *t*-test.

The intraperitoneal injection of the LPS not only induced systemic inflammation but also neuroinflammation. Regarding the hypothalamus, which is the central circadian tissue for circadian rhythm regulation, the expressions of *IL-1β*, *IL-6*, and *TNF-α* were also significantly upregulated in the LPS group at different ZTs (ZTs 3, 9, 15, and 21) over the day when compared with the control counterpart (*IL-1β*: ZT 3: *p* < 0.0001; ZT 9: *p* < 0.001; ZT 15: *p* < 0.001; ZT 21: *p* < 0.01; *IL-6*: ZT 3: *p* < 0.0001; ZT 9: *p* < 0.01; ZT 15: *p* < 0.01; ZT 21: *p* < 0.05; *TNF-α*: ZT 3: *p* < 0.001; ZT 9: *p* < 0.01; ZT 15: *p* < 0.01; ZT 21: *p* < 0.05) (Figure 2A,B,D). Interestingly, the LPS induced a moderate diurnal rhythmic expression of *IL-1β* and *IL-6* in the hypothalamus (*p* < 0.08, Table S2), while this rhythmicity was not observed in that of the control group. Considering the expression of *MCP-1*, significant upregulation was also observed in the hypothalamus of the LPS group at ZT 3 (*p* < 0.01) and ZT 21 (*p* < 0.05) when compared with the control group (Figure 2C). Additionally, LPS administration also abolished the robust diurnal oscillation of the *TNF-α* mRNA level, which is observed in the control counterpart (*p* < 0.05, Table S2 and Figure 2D).

**Figure 3:**
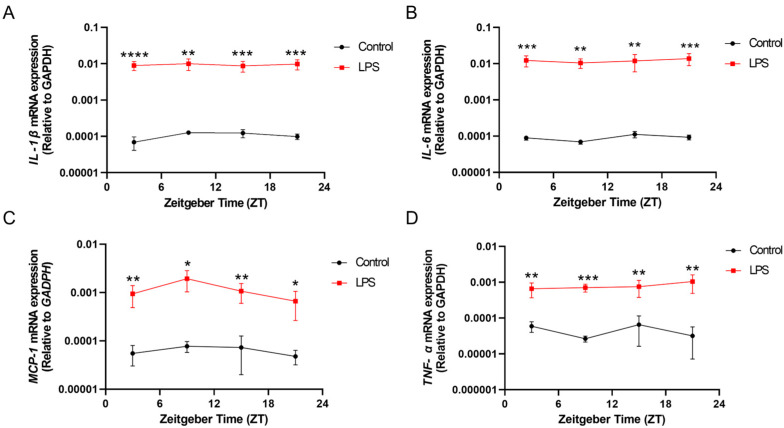
The intraperitoneal injection of the LPS induced systemic inflammation and altered the diurnal expression of proinflammatory cytokines in the hippocampus. The mRNA expressions of (**A**) *IL-1β*, (**B**) *IL-6*, (**C**) *MCP-1*, and (**D**) *TNF-α* in the hippocampus at 4 different time points (ZT 3, ZT 9, ZT 15, and ZT 21) were measured by RT-qPCR and relative to the endogenous gene *GAPDH*. Data are expressed as mean ± SEM. *n* = 3–5 mice per group for each time point. * *p* < 0.05; ** *p* < 0.01; *** *p* < 0.001; and **** *p* < 0.0001 by unpaired two-tailed Student’s *t*-test.

Systemic inflammation induced by the LPS resulted in a prominent increase in proinflammatory cytokine expressions in the hippocampus at different ZTs over the day. Significant increases were observed in *IL-1β*, *IL-6*, *MCP-1*, and *TNF-α* mRNA expression of the hippocampus of the LPS group when compared with that of the control counterpart (*IL-1β*: ZT 3: *p* < 0.0001; ZT 9: *p* < 0.01; ZT 15: *p* < 0.001; ZT 21: *p* < 0.001; *IL-6*: ZT 3: *p* < 0.001; ZT 9: *p* < 0.01; ZT 15: *p* < 0.01; ZT 21: *p* < 0.001; *MCP-1*: ZT 3: *p* < 0.01; ZT 9: *p* < 0.05; ZT 15: *p* < 0.01; ZT 21: *p* < 0.05; *TNF-α*: ZT 3: *p* < 0.01; ZT 9: *p* < 0.001; ZT 15: *p* < 0.01; ZT 21: *p* < 0.01; Figure 3A–D). Furthermore, the LPS induced a diurnal rhythmic expression of *MCP-1* in the hippocampus (*p* < 0.05, Table S3), while this rhythmicity was not observed in that of the control group.

### 2.3. Intraperitoneal Injection of LPS Induced Activation of Microglia and Perturbed Diurnal Dynamic Behavior of Microglia

**Figure 4:**
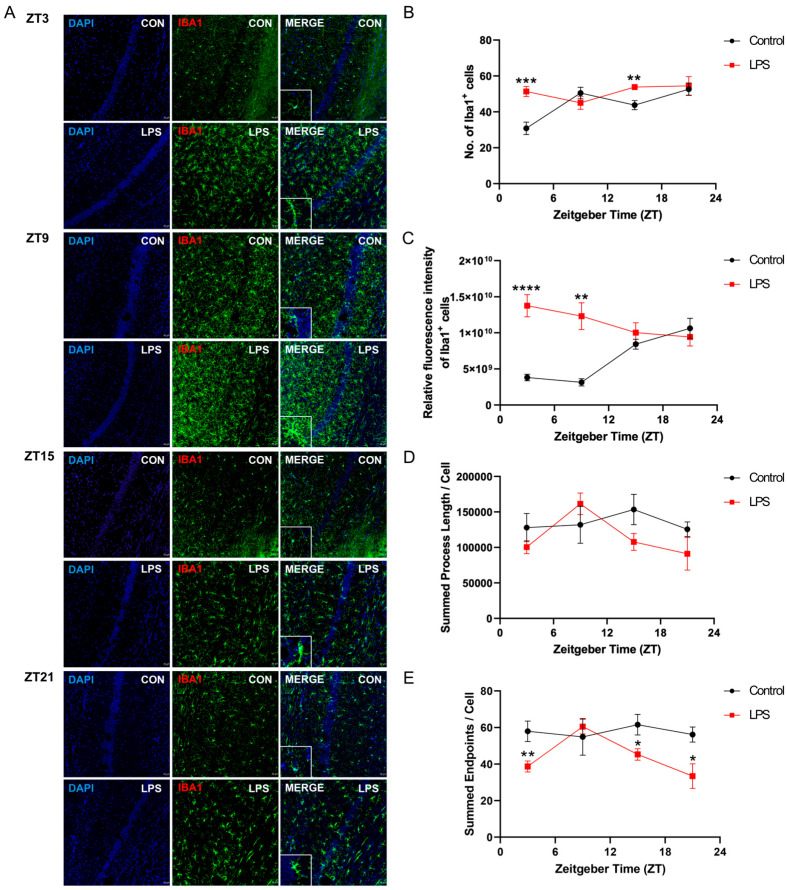
The intraperitoneal injection of the LPS induced the activation of microglia and perturbed the diurnal dynamic behavior of microglia in the CA1 of the hippocampus. (**A**) Representative confocal images of the immunohistochemical staining of DAPI and Iba1 in the CA1 of the hippocampus sections of the control and LPS groups at 4 different time points (ZT 3, ZT 9, ZT 15, and ZT 21) (Scale bar = 20 μm; 10 μm for inset). The (**B**) number of Iba1 positive cells, (**C**) the relative fluorescence intensity of Iba1 positive cells, the (**D**) summed process length per Iba1 positive cell, and the (**E**) summed endpoints per Iba1 positive cell in the CA1 of the hippocampus sections were quantified at 4 different time points (ZT 3, ZT 9, ZT 15, and ZT 21). Data are expressed as mean ± SEM. *n* = 3–5 mice per group for each time point. * *p* < 0.05; ** *p* < 0.01; *** *p* < 0.001; and **** *p* < 0.0001 by unpaired two-tailed Student’s *t*-test.

**Figure 5:**
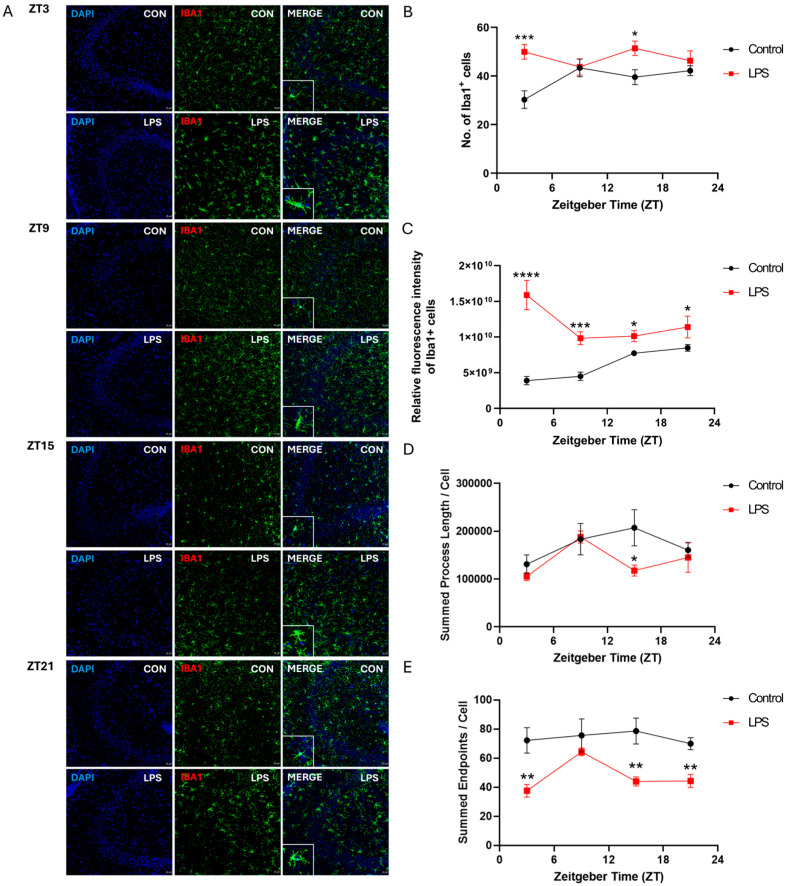
The intraperitoneal injection of the LPS induced the activation of microglia and perturbed the diurnal dynamic behavior of microglia in the CA3 of the hippocampus. (**A**) Representative confocal images of the immunohistochemical staining of DAPI and Iba1 in the CA3 of the hippocampus sections of the control and LPS groups at 4 different time points (ZT 3, ZT 9, ZT 15, and ZT 21) (Scale bar = 20 μm; 10 μm for inset). The (**B**) number of Iba1 positive cells, (**C**) the relative fluorescence intensity of Iba1 positive cells, the (**D**) summed process length per Iba1 positive cell, and the (**E**) summed endpoints per Iba1 positive cell in the CA3 of the hippocampus sections were quantified at 4 different time points (ZT 3, ZT 9, ZT 15, and ZT 21). Data are expressed as mean ± SEM. *n* = 3–5 mice per group for each time point. * *p* < 0.05; ** *p* < 0.01; *** *p* < 0.001; and **** *p* < 0.0001 by unpaired two-tailed Student’s *t*-test.

**Figure 6:**
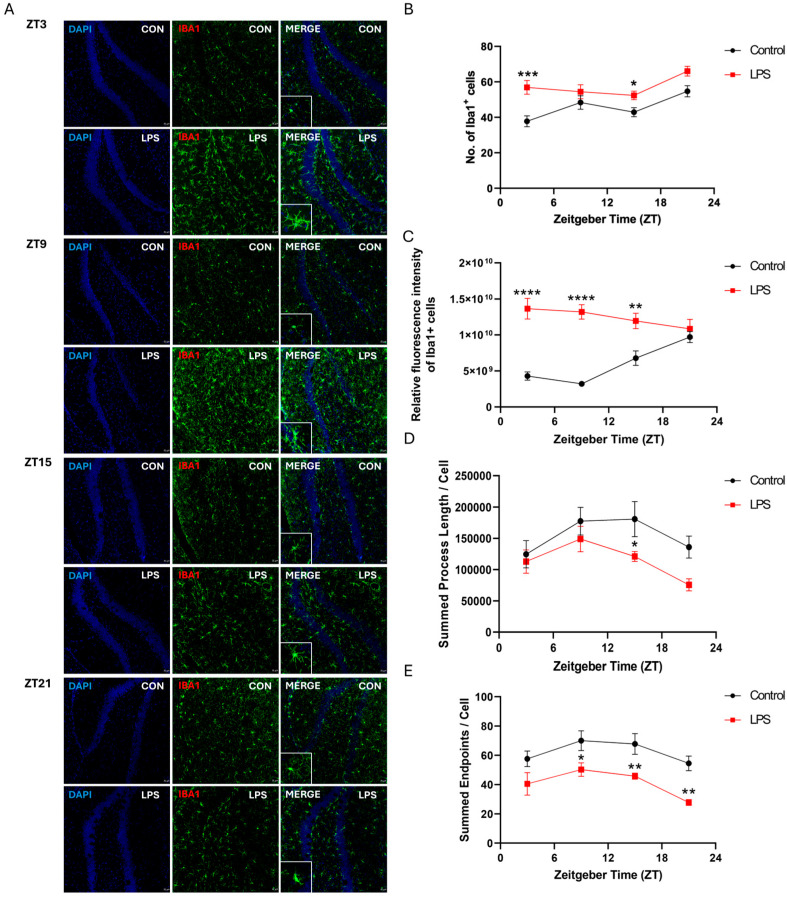
The intraperitoneal injection of the LPS induced the activation of microglia and perturbed the diurnal dynamic behavior of microglia in the DG of the hippocampus. (**A**) Representative confocal images of the immunohistochemical staining of DAPI and Iba1 in the DG of the hippocampus sections of the control and LPS groups at 4 different time points (ZT 3, ZT 9, ZT 15, and ZT 21) (Scale bar = 20 μm; 10 μm for inset). The (**B**) number of Iba1 positive cells, (**C**) the relative fluorescence intensity of Iba1 positive cells, the (**D**) summed process length per Iba1 positive cell, and the (**E**) summed endpoints per Iba1 positive cell in the DG of the hippocampus sections were quantified at 4 different time points (ZT 3, ZT 9, ZT 15, and ZT 21). Data are expressed as mean ± SEM. *n* = 3–5 mice per group for each time point. * *p* < 0.05; ** *p* < 0.01; *** *p* < 0.001; and **** *p* < 0.0001 by unpaired two-tailed Student’s *t*-test.

To investigate whether LPS affects the diurnal dynamic behavior of microglia, mouse brain sections were stained with a pan-microglial marker, ionized calcium-binding adaptor molecule 1 (Iba1). The intraperitoneal injection of an LPS perturbed the diurnal variation of the number and the fluorescent intensity of Iba1^+^ cells, as well as microglial morphological dynamics across different hippocampal subregions (Figure 4, Figure 5 and Figure 6). In the cornu ammonis (CA)1 and dentate gyrus (DG), the LPS abolished the rhythmicity in the number of microglial cells (Iba1^+^ cells), while diurnal rhythmic patterns were observed in that of the control counterpart (CA1: *p* < 0.05; DG: *p* < 0.08; Table S4 and Figure 4 and Figure 6). Moreover, a significant increase in the number of microglial cells was observed in the hippocampus of the LPS group at both ZT3 and ZT15 when compared to the control group (CA1: ZT 3: *p* < 0.001; ZT 15: *p* < 0.01; CA3: ZT 3: *p* < 0.001; ZT 15: *p* < 0.05; DG: ZT 3: *p* < 0.001; ZT 15: *p* < 0.05; Figure 4, Figure 5 and Figure 6), indicating neuroinflammation induced by systemic inflammation. Regarding the fluorescence intensity of Iba1^+^ cells, the LPS abolished rhythmicity in the expression of Iba1 in CA1 and DG, while rhythmic expression was observed in the control counterpart (*p* < 0.05, Table S4 and Figure 4 and Figure 6). Significant increases in the fluorescence intensity of Iba1^+^ cells were also observed in the CA1 and DG of the LPS group at different time points (CA1: ZT 3: *p* < 0.0001; ZT 9: *p* < 0.01; DG: ZT 3: *p* < 0.0001; ZT 9: *p* < 0.0001; ZT15: *p* < 0.01; Figure 4 and Figure 6). However, in CA3, both the LPS group and control group showed a robust rhythmic expression of Iba1 (control: *p* < 0.00001; LPS: *p* < 0.05; Table S4 and Figure 5). When compared with the control group, the LPS group showed about a 2-fold increase in the mesor of the Iba1 fluorescence intensity in the CA3, with phase advance by 16.5 h (Table S4 and Figure 5). Significant increases in the Iba1 fluorescence intensity in the CA3 were also observed throughout the day at ZTs 3, 9, 15, and 21 (ZT 3: *p* < 0.0001; ZT 9: *p* < 0.001; ZT 15: *p* < 0.05; ZT 21: *p* < 0.05; Figure 5). Regarding the microglial morphology, the microglial process length displayed rhythmicity in CA1 (*p* < 0.05), CA3 (*p* < 0.08), and DG (*p* < 0.08) of the LPS group but not the control group, with the LPS group showing a phase advance by more than 3 h (Table S4 and Figure 4, Figure 5 and Figure 6). A shortening of the microglial process length was also observed at ZT15 in both CA3 and DG of the LPS group (CA3: *p* < 0.05; DG: *p* < 0.05; Figure 5 and Figure 6). Moreover, the LPS group displayed robust rhythmicity in the number of microglial process endpoints in CA1 (*p* < 0.001) and CA3 (*p* < 0.05), while such rhythmicity was not observed in the control counterpart (Table S4 and Figure 4 and Figure 5). However, in the DG, no rhythmicity in the number of microglial process endpoints was found in both the control and LPS groups (Table S4 and Figure 6). When compared to the control counterpart, the LPS group showed less microglial process endpoints in all the hippocampal subregions, and the CA1, CA3, and DG of the LPS group over the day at different time points (*CA1*: ZT 3: *p* < 0.01; ZT 15: *p* < 0.05; ZT 21: *p* < 0.05; CA3: ZT 3: *p* < 0.01; ZT 15: *p* < 0.01; ZT 21: *p* < 0.01; DG: ZT 9: *p* < 0.05; ZT 15: *p* < 0.01; ZT 21: *p* < 0.01; Table S4 and Figure 4, Figure 5 and Figure 6). Collectively, in addition to microglial activation and deramification, these findings demonstrate that peripheral LPS administration perturbed the diurnal dynamic behavior of microglia in the hippocampus.

### 2.4. Systemic Inflammation Induced by LPS Disrupted Diurnal Oscillations of Circadian Genes in Peripheral Tissues in Liver

**Figure 7:**
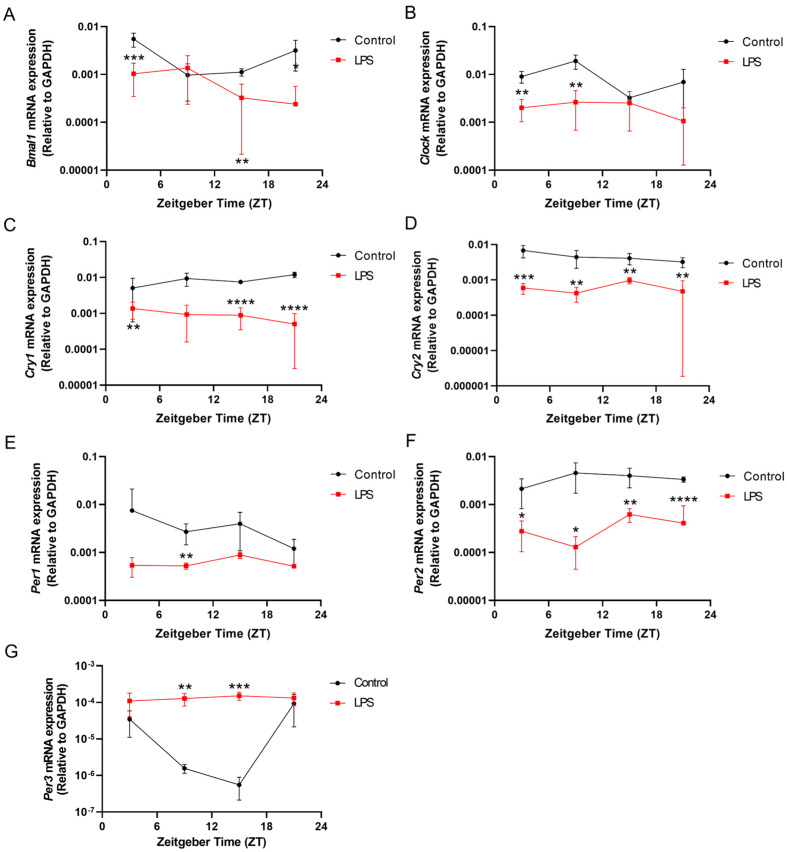
The intraperitoneal injection of the LPS disrupted the diurnal oscillations of the circadian genes in the liver. The mRNA expression of (**A**) *Bmal1*, (**B**) *Clock*, (**C**) *Cry1*, (**D**) *Cry2,* (**E**) *Per1*, (**F**) *Per2*, and (**G**) *Per3* in the liver at 4 different time points (ZT 3, ZT 9, ZT 15, and ZT 21) were measured by RT-qPCR and relative to the endogenous gene *GAPDH*. Data are expressed as mean ± SEM. *n* = 3–5 mice per group for each time point. * *p* < 0.05; ** *p* < 0.01; *** *p* < 0.001; and **** *p* < 0.0001 by unpaired two-tailed Student’s *t*-test.

Systemic inflammation induced by the LPS markedly dampened all the circadian gene expressions in the liver when compared to the control group (Figure 7). The intraperitoneal injection of the LPS reduced *Clock* mRNA expression levels in the liver at ZT 3 (*p* < 0.01) and ZT 9 (*p* < 0.01) significantly when compared with the control group (Figure 7B). The LPS also abolished the diurnal rhythmic expression of *Clock* in the liver when compared with the control counterpart (*p* < 0.05, Table S5). Regarding *Bmal1* expression in the liver, the LPS group showed significant diurnal rhythmicity (*p* < 0.05), with about a 3-fold reduction in the amplitude and phase delay compared with the control counterpart (Table S5 and Figure 7A). Significant decreases in *Bmal1* mRNA expression were also observed in the liver of the LPS group at ZT 3 and ZT 15 when compared with the control group (ZT 3: *p* < 0.001; ZT 15: *p* < 0.01) (Figure 7A). The LPS also significantly reduced the expressions of *Cry1* and *Per1* in the liver at multiple time points when compared with the control (*Cry1*: ZT 3: *p* < 0.01; ZT 15: *p* < 0.0001; ZT 21: *p* < 0.0001; *Per1*: ZT 9: *p* < 0.01) (Figure 7C,E). Meanwhile, both *Per2* and *Cry2* expression were notably downregulated in the liver of the LPS group across all time points examined—ZT 3, ZT 9, ZT 15, and ZT 21 (*Per2*: ZT 3: *p* < 0.05; ZT 9: *p* < 0.05; ZT 15: *p* < 0.01; ZT 21: *p* < 0.0001; *Cry2*: ZT 3: *p* < 0.001; ZT 9: *p* < 0.01; ZT 15: *p* < 0.01; ZT 21: *p* < 0.01) (Figure 7D,F). Furthermore, the LPS disrupted the diurnal rhythmicity of *Per3* expression in the liver, while a prominent diurnal rhythmic pattern was observed in the control counterpart (*p* < 0.05, Table S5). The LPS not only disrupted the rhythmicity but also reduced the *Per3* expression level in the liver at ZT 9 (*p* < 0.01) and ZT 15 (*p* < 0.001) significantly when compared with the control (Figure 7G).

### 2.5. Systemic Inflammation Induced by LPS Disrupted Diurnal Oscillations of Circadian Genes in Both Central Circadian Regulator in Hypothalamus and Peripheral Tissue in Hippocampus

**Figure 8:**
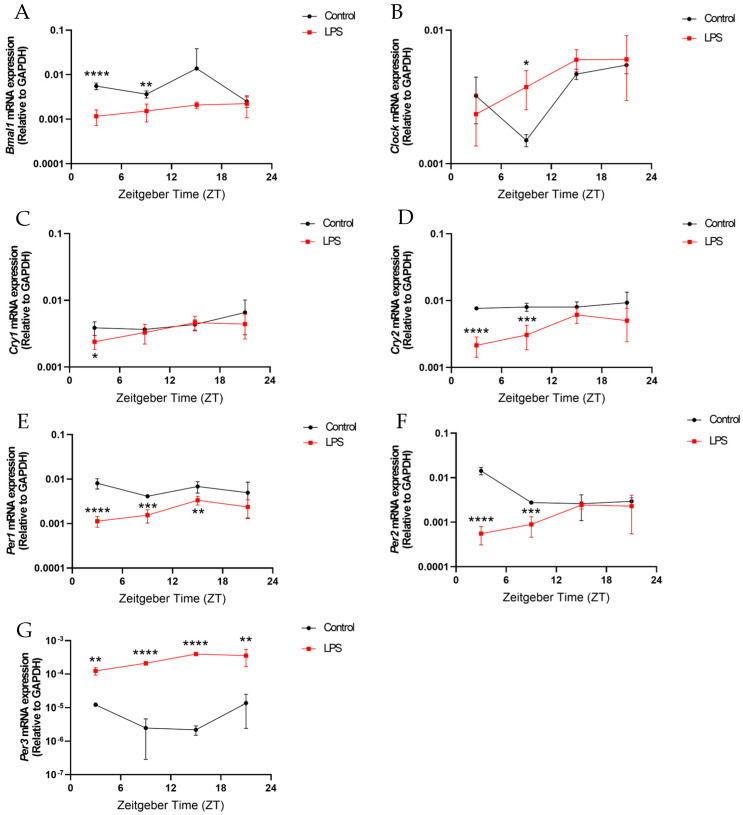
The intraperitoneal injection of an LPS disrupted the diurnal oscillations of the circadian genes in the hypothalamus. The mRNA expression of (**A**) *Bmal1*, (**B**) *Clock*, (**C**) *Cry1*, (**D**) *Cry2*, (**E**) *Per1*, (**F**) *Per2*, and (**G**) *Per3* in the hypothalamus at 4 different time points (ZT 3, ZT 9, ZT 15, and ZT 21) were measured by RT-qPCR and relative to the endogenous gene *GAPDH*. Data are expressed as mean ± SEM. *n* = 3–5 mice per group for each time point. * *p* < 0.05; ** *p* < 0.01; *** *p* < 0.001; and **** *p* < 0.0001 by unpaired two-tailed Student’s *t*-test.

Similar to the liver, the intraperitoneal injection of the LPS markedly altered the diurnal expression of circadian clock genes in the central circadian regulator, the hypothalamus (Figure 8). The LPS markedly dampened mRNA levels of all circadian genes examined, except *Clock*, in the hypothalamus at various ZTs compared to controls (*p* < 0.05, Figure 8). LPS administration led to significantly reduced mRNA expression of several core clock genes compared to the control group, including *Bmal1* at ZT3 (*p* < 0.0001) and ZT9 (*p* < 0.01), *Cry1* at ZT3 (*p* < 0.05), *Cry2* at ZT3 (*p* < 0.0001) and ZT9 (*p* < 0.001), *Per1* at ZT3 (*p* < 0.0001), ZT9 (*p* < 0.001), and ZT15 (*p* < 0.01), *Per2* at ZT3 (*p* < 0.0001) and ZT9 (*p* < 0.001), and *Per3* at ZT3 (*p* < 0.01), ZT9 (*p* < 0.0001), ZT15 (*p* < 0.0001), and ZT21 (*p* < 0.01) (Figure 8A,C–G). Moreover, *Bmal1*, *Cry1*, *Cry2*, and *Per1* all displayed significant rhythmicity in the LPS but not the control group, while for *Clock*, *Per2*, and *Per3*, their expression maintained diurnal rhythmicity in both LPS and control groups (*p* < 0.05, Table S6 and Figure 8). Overall, systemic inflammation induced by an LPS leads to aberrant circadian gene oscillations in the hypothalamus, a central circadian regulator.

**Figure 9:**
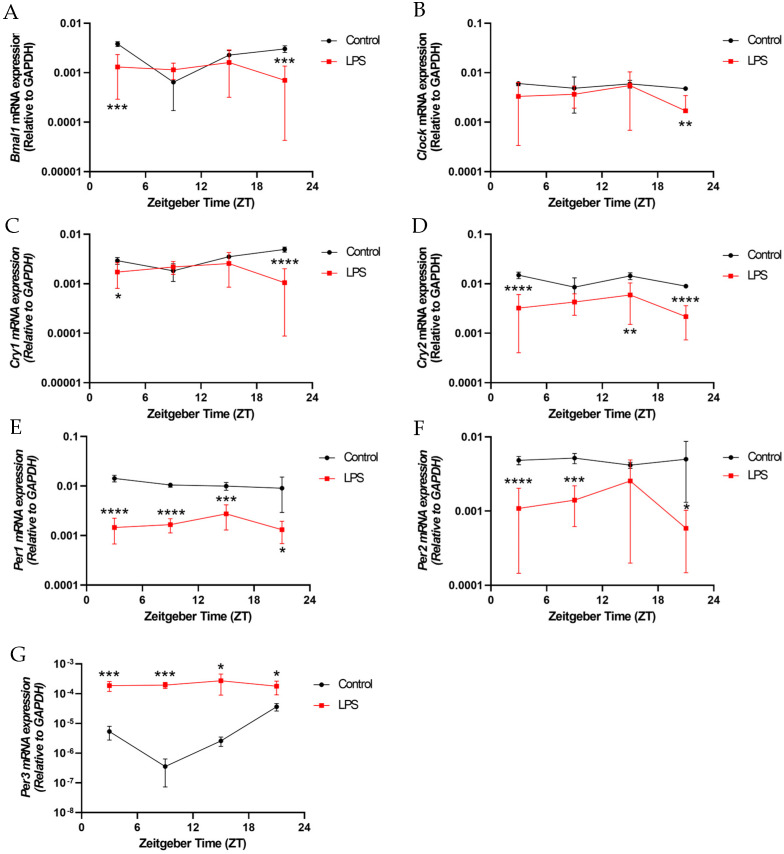
The intraperitoneal injection of the LPS disrupted the diurnal oscillations of the circadian genes in the hippocampus. The mRNA expressions of (**A**) *Bmal1*, (**B**) *Clock*, (**C**) *Cry1*, (**D**) *Cry2*, (**E**) *Per1*, (**F**) *Per2*, and (**G**) *Per3* in the hippocampus at 4 different time points (ZT 3, ZT 9, ZT 15, and ZT 21) were measured by RT-qPCR and relative to the endogenous gene *GAPDH*. Data are expressed as mean ± SEM. *n* = 3–5 mice per group for each time point. * *p* < 0.05; ** *p* < 0.01; *** *p* < 0.001; and **** *p* < 0.0001 by unpaired two-tailed Student’s *t*-test.

Intraperitoneal LPS administration disrupted the rhythmic transcription of core clock components in the hippocampus, a key circadian-regulated brain region. *Bmal1* expression in the hippocampus lacked rhythmicity in the LPS group, contrasting the prominent rhythmic pattern seen in the control group (*p* < 0.05, Table S7 and Figure 9A). Moreover, *Bmal1* expression levels were significantly reduced at ZT3 (*p* < 0.001) and ZT21 (*p* < 0.001) in the hippocampus of the LPS group compared to the control group (Figure 9A). *Clock* expression level in the hippocampus was also significantly reduced in the LPS group at ZT21 versus the control counterpart (*p* < 0.01, Figure 9B). The LPS also dampened *Cry1* expression at ZT3 (*p* < 0.05) and ZT21 (*p* < 0.0001, Figure 9C) in the hippocampus. *Cry2* expression in the hippocampus was markedly suppressed at ZT3 (*p* < 0.0001), ZT15 (*p* < 0.01), and ZT21 (*p* < 0.0001) by the injection of the LPS (Figure 9D). The LPS additionally led to reduced *Per1*, *Per2*, and *Per3* expression levels in the hippocampus at different ZTs versus the control group. (*Per1*: ZT3: *p* < 0.0001, ZT9: *p* < 0.0001; ZT15: *p* < 0.001; ZT21: *p* < 0.05; *Per2*: ZT3: *p* < 0.0001, ZT9: *p* < 0.001; ZT21: *p* < 0.05; *Per3*: ZT3: *p* < 0.001, ZT9: *p* < 0.001; ZT15: *p* < 0.05; ZT21: *p* < 0.05) (Figure 9E–G). Furthermore, *Per3* expression in the hippocampus displayed rhythmicity in the control group (*p* < 0.05) but not in the LPS group (Table S7 and Figure 9G). Altogether, these results demonstrate that peripheral LPS-induced inflammation broadly disturbs the diurnal expression of multiple core circadian genes in the hippocampus.

## 3. Discussion

Circadian rhythms govern multiple physiological processes, and their disruption is linked to various pathological conditions. Factors like inflammation may perturb circadian rhythms, though these interactions remain unclear. Here, our study revealed that systemic inflammation induced by a single intraperitoneal injection of an LPS perturbed diurnal oscillations of circadian genes in both the central circadian regulator in the hypothalamus and the peripheral tissues in the hippocampus and liver, suggesting a validated model for investigating the effects of systemic inflammation on circadian desynchrony. Furthermore, we demonstrated that systemic inflammation could induce neuroinflammation and perturb the diurnal dynamic behavior of microglia, emphasizing the close interactions between inflammation and circadian regulation. Our model provides a useful tool to elucidate the mechanisms by which systemic inflammation impacts central and peripheral circadian rhythms and associated immune responses.

Our study provided evidence that systemic inflammation is closely linked to circadian rhythms. Systemic inflammation induced by the intraperitoneal injection of the LPS altered the diurnal oscillations of the circadian genes in the hypothalamus, hippocampus, and liver (Figure 7, Figure 8 and Figure 9). It is proposed that systemic inflammation potentially affects the pacemaker of circadian rhythm, the SCN in the hypothalamus. A previous study has revealed that systemic inflammation significantly reduced light-induced Fos expression in the SCN, indicating that inflammation could alter SCN photic responsiveness [31]. Similarly, our findings showed that LPS administration perturbed the rhythmic expression of circadian genes under a normal light–dark cycle.

Inflammation induced by the LPS perturbed the rhythmic expression of *MCP-1* in both the liver and hippocampus (Figure 1 and Figure 3 and Tables S1 and S3), while previous work has shown that *rev-erbα*, a component of the accessory circadian feedback loop, directly regulates the inflammatory functions of macrophages through regulation of *MCP-1* expression [32]. As *MCP-1* plays a critical role in recruiting monocytes to the inflammation sites, our results suggest that inflammation-dysregulated *MCP-1* rhythms may in turn affect macrophage infiltration into the brain, potentially influencing the development of neuroinflammatory processes.

Our findings newly demonstrate that the diurnal dynamic variation of microglia was perturbed by the LPS. In hippocampal subregions CA1 and DG, the LPS abolished rhythmicity in microglial cell numbers that were present in the control group (Figure 4 and Figure 6 and Table S4). Additionally, the LPS abolished rhythmic Iba1 fluorescence intensity in CA1 and DG, while rhythmicity was observed in the control counterpart (Figure 4 and Figure 6 and Table S4). Meanwhile, the LPS perturbed the diurnal behavior of microglia. The LPS induced rhythmicity in the microglial process length in CA1, CA3, and DG where controls showed arrhythmicity (Figure 4, Figure 5 and Figure 6 and Table S4). The LPS additionally imposed rhythmicity in process endpoints in CA1 and CA3 that were absent in the control group (Figure 4 and Figure 5 and Table S4). Furthermore, the LPS resulted in phase advances of the microglial process length and endpoints in all the hippocampal subregions, such that the acrophase occurred during the light rather than the dark phase. This contrasted with the control group, which showed peak process length and endpoints during the dark (active period). This matches prior work showing increased cortical microglial branching and process extension in the wild-type control mice during the active phase (dark) [33]. Furthermore, the LPS induced microglial deramification, as evidenced by reduced process length and endpoints specifically during the dark phase (Figure 4, Figure 5 and Figure 6). Overall, our study reveals that the LPS dysregulates hippocampal microglial diurnal dynamics throughout the 24 h period; meanwhile, we also defined microglial diurnal behavior in the normal physiological state, providing insights into how systemic inflammation disrupts the temporal coordination of microglial immune surveillance.

In addition to perturbing circadian rhythms, our findings demonstrate that systemic inflammation could also provoke neuroinflammatory responses, as evidenced by the increase in the proinflammatory cytokine expression (Figure 1, Figure 2 and Figure 3) and also microgliosis (Figure 4, Figure 5 and Figure 6). These findings align with our previous study that used different models for inducing systemic inflammation [27,28,29]. Over a lifetime, there are occasional encounters with systemic inflammation induced by infections, injuries, or other illnesses. This can interfere with circadian rhythms and promote neuroinflammation. Our studies showed that systemic inflammation could disrupt the diurnal rhythms of central and peripheral circadian tissues, and also perturb the diurnal behavior of hippocampal microglia, potentially desynchronizing oscillators that regulate inflammatory processes. Such circadian dysregulation may exacerbate neuroinflammation and the pathogenesis of neurodegeneration. Further studies elucidating how circadian clock machinery regulates inflammatory pathways in microglia and other immune cells would provide valuable insights into the connection between circadian rhythms and neuroinflammation.

## 4. Materials and Methods

### 4.1. Animal Housing

Male wild-type C57BL/6J mice (8–10 weeks) were provided by the Centre for Comparative Medicine Research (CCMR) of the University of Hong Kong. All procedures involving the animals were approved by the faculty committee on the Use of Live Animals in Teaching and Research (CULATR) of the University of Hong Kong (#5197-19) and the Department of Health of the Hong Kong SAR government. Mice were housed under constant environmental settings, with a temperature controlled at 20–22 °C, a humidity of 50–60%, a light intensity of 200–300 lux, and a 12 h light/12 h dark cycle. All mice had unlimited access to food and water. Mice were given 2 weeks to habituate to the environmental conditions prior to the experiments.

### 4.2. Experimental Protocols

Mice were randomly assigned to two groups: the saline control group and the LPS group. On day 0, the control group received an intraperitoneal injection of saline, while mice in the LPS group were administered an intraperitoneal injection of an LPS from *Escherichia coli* (LPS25, Sigma-Aldrich, St. Louis, MO, USA; O111:B4) at a dose of 5 mg/kg body weight, at ZT 8. On day 2 post-injection, mice were euthanized via carbon dioxide asphyxiation followed by decapitation at 6 h intervals throughout the day. Mice were then transcardially perfused with ice-cold 0.9% saline.

### 4.3. RNA Extraction and Quantitative Reverse Transcription Polymerase Chain Reaction (RT-qPCR) Analysis

Tissues including the liver, hippocampus, and hypothalamus were harvested and snap-frozen in liquid nitrogen and then stored at −80 °C. The total RNA of the harvested tissues was extracted using RNAiso Plus (TAKARA, Shiga, Japan) according to the manufacturer’s protocol. The purity and concentration of RNA samples were measured using NanoDrop light (Thermo Fisher Scientific, Waltham, MA, USA). The complementary DNA (cDNA) was then synthesized from the purified RNA using PrimeScript^TM^ RT Master Mix (TAKARA, Japan) following the manufacturer’s protocol. qPCR was performed using iTaq Universal SYBR Green Supermix (Bio-Rad Laboratories, Hercules, CA, USA) and Roche LightCycler 480 II (Roche, Switzerland). The primer sequences utilized are listed in Table S8. The relative expression levels were determined using the comparative CT method to normalize the target gene mRNA to GAPDH.

### 4.4. Tissue Preparation for Cryosection

The procedures for preparing cryosections were similar to the steps for preparing human brain sections described in our previous study [34]. After post-fixation in 4% paraformaldehyde (PFA) for 24 h, the brain samples were immersed in 20% sucrose solution overnight followed by 30% sucrose solution at 4 °C. The tissues were then embedded in an optimal cutting temperature (OCT) compound and sectioned using a Leica CM1950-cryotast (Leica Biosystems, Nußloch, Germany). Then, 20 μm thick coronal sections were mounted onto glass slides. The prepared slides were stored at −80 °C until immunostaining.

### 4.5. Immunohistochemistry (IHC) Staining

Prior to confocal imaging, IHC staining was performed as described previously [27]. The sample sections were blocked with 10% donkey serum (D9663, Sigma-Aldrich, USA) for 1 h. The sections were then incubated overnight at 4 °C with the following primary antibody diluted at 1:400 in 0.3% Triton-X: rabbit anti-Iba1 antibody (SKM6526, FUJIFILM Wako, Tokyo, Japan). After washing three times with PBS, the sections were then incubated for 1 h in the dark with Alexa Fluor^®^ 488-conjugated goat anti-rabbit secondary antibody (Invitrogen, Carlsbad, CA, USA) at a 1:800 dilution. The sections were washed three more times with PBS, and then stained with 4′,6-diamidino-2-2phenylindole (DAPI). All section slides were mounted using a fluorescence mounting medium (CS703, Dako, Santa Clara, CA, USA) and stored in the dark at 4 °C until confocal imaging.

### 4.6. Confocal Microscopy

Confocal images were acquired using a Leica TCS SP8 MP multiphoton/confocal microscope (Leica Biosystem, Germany) with a 20× objective lens at 1024 × 1024 resolution using LAS X software (Version 5.2.2). Z-stack images were acquired from three hippocampal regions—the DG, CA1, and CA3. Orthogonal projections were generated from the Z-stack images using LAS X software. ImageJ software (version 1.54j, National Institute of Health, USA) was used to analyze the images. At least 3 sections of each brain region per animal were examined for qualitative and quantitative analyses. The fluorescence intensity was quantified by calculating the corrected total cell fluorescence (CTCF) using the following formula: Integrated Density—(Area of Selected Cell × Mean Fluorescence of Background Readings). Skeleton analyses on microglial morphology were performed on ImageJ (National Institute of Health, Bethesda, MD, USA) as described previously [35].

### 4.7. Statistical Analysis

Unless otherwise specified, all statistical analyses were performed on GraphPad Prism v8.0c (GraphPad Software, Boston, MA, USA). Data were expressed as means ± SEM. The normality of data was assessed using the D’Agostino–Pearson normality test. An unpaired two-tailed Student’s *t*-test at *p* < 0.05 was applied to compare the two groups to determine the statistical significance of the effects of LPS treatment. CircWave software (version 1.4) was used to analyze the presence of diurnal rhythmicity. Mesor, amplitude, and acrophase were calculated using cosinor analysis (CosinorOnline).

## 5. Conclusions

In summary, our findings demonstrated that intraperitoneal injection of the LPS profoundly perturbed the diurnal expressions of circadian genes in both central and peripheral tissues. Systemic inflammation induced by peripheral LPS administration could not only induce neuroinflammation but also perturb diurnal behavioral changes in the hippocampal microglia. Elucidating the intricate bidirectional relationships between inflammation and circadian rhythms may ultimately uncover mechanisms to prevent systemic inflammation-induced neuroinflammation and neurodegeneration.

## Data Availability

The original contributions presented in this study are included in the article/Supplementary Materials. Further inquiries can be directed to the corresponding authors.

## Supplementary Materials

The supporting information can be downloaded at: https://www.mdpi.com/article/10.3390/ijms25137458/s1.
