# Supplementary material for: Systemic Inflammation Disrupts Circadian Rhythms and Diurnal Neuroimmune Dynamics
Source: Int J Mol Sci. 2024 Jul 7;25(13):7458. doi: 10.3390/ijms25137458 (PMC11242289; doi:10.3390/ijms25137458)
Supplement: Supplementary file 1 [file ijms-25-07458-s001.zip › ijms-3044246-supplementary.pdf]

**Supplementary information**

Table S1. Cosinor analysis of proinflammatory cytokine expressions in the liver.

| Tissue | Gene | Group | Mesor | Amplitude | Acrophase | *p*-value |
|---|---|---|---|---|---|---|
| Liver | *IL-1β* | Control | $3.6 \times 10^{-03}$ | $2.7 \times 10^{-03}$ | 8.0 | 0.109 |
| | | LPS | $1.8 \times 10^{-02}$ | $5.7 \times 10^{-03}$ | 9.6 | 0.241 |
| | *IL-6* | Control | $3.2 \times 10^{-04}$ | $8.3 \times 10^{-05}$ | 7.0 | 0.922 |
| | | LPS | $1.4 \times 10^{-02}$ | $1.7 \times 10^{-03}$ | 3.9 | 0.243 |
| | *TNF-α* | Control | $2.6 \times 10^{-04}$ | $7.5 \times 10^{-05}$ | 23.9 | 0.622 |
| | | LPS | $2.0 \times 10^{-03}$ | $2.9 \times 10^{-04}$ | 5.4 | 0.554 |
| | *MCP-1* | Control | $4.8 \times 10^{-03}$ | $7.8 \times 10^{-03}$ | 8.9 | 0.005 |
| | | LPS | $1.3 \times 10^{-02}$ | $6.6 \times 10^{-03}$ | 8.7 | 0.288 |

Notes: $p < 0.05$; $p < 0.05$ indicates the presence of diurnal rhythmicity of the proinflammatory cytokine expressions.

Table S2. Cosinor analysis of proinflammatory cytokine expressions in the hypothalamus.

| Tissue | Gene | Group | Mesor | Amplitude | Acrophase | *p*-value |
|---|---|---|---|---|---|---|
| **Hypothalamus** | *IL-1β* | Control | $2.4 \times 10^{-04}$ | $1.5 \times 10^{-04}$ | 20.5 | 0.162 |
| | | LPS | $7.0 \times 10^{-03}$ | $1.5 \times 10^{-03}$ | 6.2 | 0.060 |
| | *IL-6* | Control | $1.6 \times 10^{-04}$ | $9.2 \times 10^{-05}$ | 11.9 | 0.217 |
| | | LPS | $7.5 \times 10^{-03}$ | $2.3 \times 10^{-03}$ | 4.7 | 0.068 |
| | *TNF-α* | Control | $8.3 \times 10^{-03}$ | $1.4 \times 10^{-02}$ | 14.6 | 0.003 |
| | | LPS | $1.5 \times 10^{-03}$ | $5.3 \times 10^{-04}$ | 9.1 | 0.681 |
| | *MCP-1* | Control | $8.0 \times 10^{-05}$ | $5.8 \times 10^{-05}$ | 7.4 | 0.154 |
| | | LPS | $7.0 \times 10^{-04}$ | $9.9 \times 10^{-05}$ | 12.8 | 0.420 |

Notes: $p < 0.05$; $p < 0.08$. Both $p < 0.05$ and $p < 0.08$ indicate the presence of diurnal rhythmicity of the proinflammatory cytokine expressions.

Table S3. Cosinor analysis of proinflammatory cytokine expressions in the hippocampus.

| Tissue | Gene | Group | Mesor | Amplitude | Acrophase | *p*-value |
|---|---|---|---|---|---|---|
| **Hippocampus** | *IL-1β* | Control | $9.0x10^{-05}$ | $1.8x10^{-05}$ | 17.5 | 0.497 |
| | | LPS | $9.3x10^{-03}$ | $1.6x10^{-04}$ | 5.8 | 0.985 |
| | *IL-6* | Control | $9.0x10^{-05}$ | $1.4x10^{-05}$ | 17.8 | 0.135 |
| | | LPS | $1.2x10^{-02}$ | $1.7x10^{-03}$ | 21.4 | 0.520 |
| | *TNF-α* | Control | $6.0x10^{-05}$ | $1.7x10^{-05}$ | 11.1 | 0.982 |
| | | LPS | $1.1x10^{-03}$ | $6.2x10^{-04}$ | 9.4 | 0.363 |
| | *MCP-1* | Control | $5.0x10^{-05}$ | 2.2x10-06 | 11.6 | 0.344 |
| | | LPS | $7.9x10^{-04}$ | $1.7x10^{-04}$ | 20.0 | 0.017 |

Notes: $p < 0.05$; $p < 0.08$. Both $p < 0.05$ and $p < 0.08$ indicate the presence of diurnal rhythmicity of the proinflammatory cytokine expressions.

Table S4. Cosinor analysis of spatiotemporal characteristics of $Iba1^{+}$ cells in each brain region.

| | Brain region | Treatment | Mesor | Amplitude | Acrophase | *p*-value |
|---|---|---|---|---|---|---|
| No. of $Iba1^{+}$ cells | CA1 | Control | 43.5 | 8.3 | 16.4 | 0.022 |
| | | LPS | 51.5 | 5.3 | 20.4 | 0.168 |
| | CA3 | Control | 38.4 | 5.4 | 15.5 | 0.086 |
| | | LPS | 48.7 | 2.3 | 20.4 | 0.736 |
| | DG | Control | 45.3 | 6.2 | 18.6 | 0.079 |
| | | LPS | 56.5 | 5.2 | 22.5 | 0.254 |
| Relative fluorescence intensity of $Iba1^{+}$ cells | CA1 | Control | 64523.2 | 45252.1 | 18.9 | <0.00001 |
| | | LPS | 114918.8 | 23645.4 | 5.2 | 0.164 |
| | CA3 | Control | 61111.8 | 28923.4 | 18.1 | <0.00001 |
| | | LPS | 121593.9 | 32724.2 | 1.6 | 0.029 |
| | DG | Control | 59531.2 | 36496.5 | 19.6 | <0.00001 |
| | | LPS | 125335.8 | 13615.7 | 6.3 | 0.405 |
| Summed process length/ cell | CA1 | Control | 135389.2 | 12652.9 | 13.5 | 0.640 |
| | | LPS | 112691.6 | 39433.7 | 9.6 | 0.005 |
| | CA3 | Control | 170435.2 | 39889.3 | 14.0 | 0.081 |
| | | LPS | 130699.3 | 32823.5 | 10.2 | 0.055 |
| | DG | Control | 154367.6 | 34889.2 | 12.7 | 0.107 |
| | | LPS | 115341.0 | 35985.4 | 9.4 | 0.068 |
| Summed endpoints/ cell | CA1 | Control | 57.9 | 1.4 | 15.1 | 0.939 |
| | | LPS | 43.9 | 14.9 | 10.0 | 0.001 |
| | CA3 | Control | 74.2 | 4.4 | 11.8 | 0.756 |
| | | LPS | 45.4 | 13.1 | 10.2 | 0.007 |
| | DG | Control | 63.0 | 9.3 | 11.2 | 0.120 |
| | | LPS | 42.0 | 10.7 | 10.0 | 0.144 |

Notes: $p < 0.05$; $p < 0.08$. Both $p < 0.05$ and $p < 0.08$ indicate the presence of diurnal rhythmicity.

Table S5. Cosinor analysis of circadian genes in the liver.

| Tissue | Gene | Group | Mesor | Amplitude | Acrophase | *p*-value |
|---|---|---|---|---|---|---|
| Liver | *Bmal1* | Control | $2.8 \times 10^{-03}$ | $2.4 \times 10^{-03}$ | 1.5 | 0.002 |
| | | LPS | $7.4 \times 10^{-04}$ | $6.6 \times 10^{-04}$ | 6.9 | 0.044 |
| | *Clock* | Control | $9.3 \times 10^{-03}$ | $5.7 \times 10^{-03}$ | 7.2 | 0.035 |
| | | LPS | $2.1 \times 10^{-03}$ | $7.7 \times 10^{-04}$ | 10.3 | 0.403 |
| | *Cry1* | Control | $8.3 \times 10^{-03}$ | $2.4 \times 10^{-03}$ | 18.5 | 0.240 |
| | | LPS | $9.2 \times 10^{-04}$ | $3.2 \times 10^{-04}$ | 5.4 | 0.373 |
| | *Cry2* | Control | $4.7 \times 10^{-03}$ | $1.6 \times 10^{-03}$ | 5.1 | 0.123 |
| | | LPS | $6.1 \times 10^{-04}$ | $1.8 \times 10^{-04}$ | 16.0 | 0.295 |
| | *Per1* | Control | $4.0 \times 10^{-03}$ | $2.3 \times 10^{-03}$ | 5.2 | 0.680 |
| | | LPS | $6.2 \times 10^{-04}$ | $1.6 \times 10^{-04}$ | 15.5 | 0.054 |
| | *Per2* | Control | $3.5 \times 10^{-03}$ | $1.1 \times 10^{-03}$ | 13.1 | 0.227 |
| | | LPS | $3.6 \times 10^{-04}$ | $2.2 \times 10^{-04}$ | 17.9 | 0.110 |
| | *Per3* | Control | $3.0 \times 10^{-05}$ | $5.2 \times 10^{-05}$ | 22.5 | 0.025 |
| | | LPS | $1.3 \times 10^{-04}$ | $2.1 \times 10^{-05}$ | 15.4 | 0.490 |

Notes: $p < 0.05$; $p < 0.08$. Both $p < 0.05$ and $p < 0.08$ indicate the presence of diurnal rhythmicity of the circadian gene.

Table S6. Cosinor analysis of circadian genes in the hypothalamus.

| Tissue | Gene | Group | Mesor | Amplitude | Acrophase | *p*-value |
|---|---|---|---|---|---|---|
| **Hypothalamus** | *Bmal1* | Control | $1.8x10^{-03}$ | $5.8x10^{-04}$ | 17.5 | 0.615 |
| | | LPS | $9.7x10^{-04}$ | $3.9x10^{-04}$ | 3.6 | 0.064 |
| | *Clock* | Control | $3.7x10\text{-}^{03}$ | $2.1x10^{-03}$ | 19.6 | 0.005 |
| | | LPS | $4.5x10^{-03}$ | $2.2x10^{-03}$ | 17.1 | 0.006 |
| | *Cry1* | Control | $4.6x10^{-03}$ | $1.6x10^{-03}$ | 20.2 | 0.138 |
| | | LPS | $3.7x10^{-03}$ | $1.2x10^{-03}$ | 16.8 | 0.015 |
| | *Cry2* | Control | $8.2x10^{-03}$ | $8.9x10^{-04}$ | 20.0 | 0.656 |
| | | LPS | $4.1x10^{-03}$ | $2.2x10^{-03}$ | 16.7 | 0.002 |
| | *Per1* | Control | $6.1x10^{-03}$ | $7.9x10^{-04}$ | 2.9 | 0.722 |
| | | LPS | $2.1x10^{-03}$ | $1.2x10^{-03}$ | 16.3 | 0.000 |
| | *Per2* | Control | $5.9x10^{-03}$ | $6.1x10^{-03}$ | 3.4 | 0.000 |
| | | LPS | $1.5x10^{-03}$ | $1.2x10^{-03}$ | 17.5 | 0.003 |
| | *Per3* | Control | $1.0x10^{-05}$ | $7.6x10^{-06}$ | 23.8 | 0.021 |
| | | LPS | $2.7x10^{-04}$ | $1.5x10^{-04}$ | 16.9 | 0.000 |

Notes: $p < 0.05$; $p < 0.08$. Both $p < 0.05$ and $p < 0.08$ indicate the presence of diurnal rhythmicity of the circadian gene.

Table S7. Cosinor analysis of circadian genes in the hippocampus.

| Tissue | Gene | Group | Mesor | Amplitude | Acrophase | *p*-value |
|---|---|---|---|---|---|---|
| **Hippocampus** | *Bmal1* | Control | $1.2x10^{-03}$ | $2.7x10^{-04}$ | 11.3 | 0.001 |
| | | LPS | $1.1x10^{-03}$ | $1.1x10^{-04}$ | 16.6 | 0.670 |
| | *Clock* | Control | $5.5x10^{-03}$ | $2.2x10^{-04}$ | 7.1 | 0.921 |
| | | LPS | $3.6x10^{-03}$ | $1.5x10^{-03}$ | 12.2 | 0.357 |
| | *Cry1* | Control | $3.3x10^{-03}$ | $1.6x10^{-03}$ | 20.3 | 0.005 |
| | | LPS | $1.9x10^{-03}$ | $7.1x10^{-04}$ | 11.4 | 0.176 |
| | *Cry2* | Control | $1.2x10^{-02}$ | $6.9x10^{-04}$ | 7.5 | 0.886 |
| | | LPS | $3.9x10^{-03}$ | $1.7x10^{-03}$ | 12.5 | 0.203 |
| | *Per1* | Control | $1.1x10^{-02}$ | $2.5x10^{-03}$ | 4.6 | 0.185 |
| | | LPS | $1.8x10^{-03}$ | $6.6x10^{-04}$ | 14.0 | 0.119 |
| | *Per2* | Control | $4.8x10^{-03}$ | $3.1x10^{-04}$ | 3.0 | 0.906 |
| | | LPS | $1.4x10^{-03}$ | $8.4x10^{-04}$ | 13.0 | 0.187 |
| | *Per3* | Control | $1.0x10^{-05}$ | $1.6x10^{-05}$ | 21.2 | 0.005 |
| | | LPS | $2.1x10^{-04}$ | $4.5x10^{-05}$ | 14.3 | 0.457 |

Notes: $p < 0.05$; $p < 0.08$. Both $p < 0.05$ and $p < 0.08$ indicate the presence of diurnal rhythmicity of the circadian gene.

Table S8. Sequences of primers for RT-qPCR.

| Gene name | | Primer sequences |
|---|---|---|
| *Bmal1* | Forward | 5′-ATTCCAGGGGGAACCAGA-3′ |
| | Reverse | 5′-GAAGGTGATGACCCTCTTATCCT-3′ |
| *Clock* | Forward | 5′-CACAGGCCAGCACATGAT-3′ |
| | Reverse | 5′-CACTCATTACACTCTGTTGACTCTGA-3′ |
| *Cry1* | Forward | 5′-ATCGTGCGCATTTCACATAC-3′ |
| | Reverse | 5′-TCCGCCATTGAGTTCTATGAT-3′ |
| *Cry2* | Forward | 5′-GGGAGCATCAGCAACACAG-3′ |
| | Reverse | 5′-GCTTCCAGCTTGCGTTTG-3′ |
| *Per1* | Forward | 5′-GTGGGCTTGACACCTCTTCT-3′ |
| | Reverse | 5′-TGCTTTAGATCGGCAGTGGT-3′ |
| *Per2* | Forward | 5′-GTTCCAGGCTGTGGATGAA-3′ |
| | Reverse | 5′-GGCGTCTCGATCAGATCCT-3′ |
| *Per3* | Forward | 5′-AAAAGCACCACGGATACTGGC-3′, |
| | Reverse | 5′-GGGACCCTGTAGCTTGTC-3′ |
| *IL-1β* | Forward | 5’ GATGAAGGGCTGCTTCCAAAC-3’ |
| | Reverse | 5’-TCCACAGCCACAATGAGTGA-3’ |
| *IL-6* | Forward | 5’-TTCACAAGTCCGGAGAGGAG-3’ |
| | Reverse | 5’TCCACGATTTCCCAGAGAAC-3’ |
| *MCP-1* | Forward | 5’-TGCTGTCTCAGCCAGATGCAGTTA-3’ |
| | Reverse | 5’-TACAGCTTCTTTGGGACACCTGCT-3’ |
| *TNF-α* | Forward | 5’ CCCCAGTCTGTATCCTCCT-3’ |
| | Reverse | 5’ ACTGTCCCAGCATCTTGT-3’ |
| *GAPDH* | Forward | 5’-ATTCAACGGCACAGTCAA-3’ |
| | Reverse | 5’-CTCGCTCCTGGAAGATGG-3’ |
